# Supplementary material for: Exploring barriers and enablers to diabetes self-care practice in Ethiopia, 2025: A qualitative systematic review
Source: PLoS One. 2026 Apr 10;21(4):e0346867. doi: 10.1371/journal.pone.0346867 (PMC13068285; doi:10.1371/journal.pone.0346867)
Supplement: S1 File — (DOCX) [file pone.0346867.s001.docx]

**S1. List of articles excluded in the final studies Exploring Barriers and Enablers to Diabetes Self-Care in Ethiopia, 2025**

| Author and year of publication | Tittle | Remark | Reason |
| --- | --- | --- | --- |
| Abate, T. W. et.al,2021 | Non-adherence to self-care and associated factors among diabetes adult population in Ethiopian: A systemic review with meta-analysis | Excluded | It is reviewing paper not original article |
| Ketema, D.B,et.al,2020 | Level of self-care practice among diabetic patients in Ethiopia: a systematic review and meta-analysis | Excluded | It is reviewing paper not original article and focus on quantative |
| Hailu, F. B,2019 | Diabetes Self-Management Education (DSME) – Effect on Knowledge, Self-Care Behavior, and Self-Efficacy Among Type 2 Diabetes Patients in Ethiopia: A Controlled Clinical Trial | Excluded | Focus on effect of education rather than barriers and facilitators toward diabetic’s self-care practice |
| Kassahun, T. et.al 2016 | Diabetes related knowledge, self-care behaviours and adherence to medications among diabetic patients in Southwest Ethiopia: a cross-sectional survey | Excluded | Focus on medication adherence rather than barriers and facilitators toward diabetic’s self-care practice |
| Zeleke.et.al,2018 | Adherence to diabetic self-care practices and its associated factors among patients with type 2 diabetes in Addis Ababa, Ethiopia | Excluded | It focusses on adherence |
| Mariye, T,et.al,2017 | Magnitude of diabetes self-care practice and associated factors among type two adult diabetic patients following at public Hospitals in central zone, Tigray Region, Ethiopia, 2017 | Excluded | It focusses on magnitudes rather than barriers and facilitators toward diabetic’s self-care practice |
| Niguse, H.et.al,2019 | Self-care related knowledge, attitude, practice and associated factors among patients with diabetes in Ayder Comprehensive Specialized Hospital, North Ethiopia | Excluded | It focusses on KAP rather than barriers and facilitators toward diabetic’s self-care practice |
| Semegn,et.al.2023 | Adherence to Self - Care Practice Among Type 2 Diabetes Mellitus Patients Using the Theory of Planned Behavior and Health Belief Model at Comprehensive Specialized Hospitals of Amhara Region, Ethiopia: Mixed Method | Excluded | It focusses on adherence rather than barriers and facilitators toward diabetic’s self-care practice |
| Yehualashet, F.A.et.al,2024 | Feasibility of diabetes self-management coaching program for individuals with type 2 diabetes in the Ethiopian primary care setting: a protocol for a feasibility mixed-methods parallel-group randomized controlled trial | Excluded | It focusses on feasibility of self-care rather than barriers and facilitators toward diabetic’s self-care practice |
| Diriba, D. C.et.al,2021 | A nurse-led, community-based self-management program for people living with type 2 diabetes in Western Ethiopia: A feasibility and pilot study protocol | Excluded | Wrong population |
| Diriba, D. C.et.al,2023 | Effects of family-based diabetes self-management education and support programme on support behaviour amongst adults with type 2 diabetes in Western Ethiopia | Excluded | Focus on effects of family education and their supports rather than barriers and facilitators toward diabetic’s self-care practice |
| Hailu, F. B,2018 | Nurse-Led Diabetes Self-Management Education Improves Clinical Parameters in Ethiopia | Excluded | Wrong poulation |
| Habebo ,T,T. et..al,2020 | Prevalence of Poor Diabetes Self-Management Behaviors among Ethiopian Diabetes Mellitus Patients: A Systematic Review and Meta- Analysis | Excluded | Focus on quantative |
| Gurmu Y and Abebe Dechasa,2023 | Effect of patient centered diabetes self care management education among adult diabetes patients in Ambo town, Ethiopia: An interventional study | Excluded | Focus on Effect of patient centered education |
| Mekonen, E.G,et.l,2021 | Knowledge, attitude, and practice towards self-management among diabetic patients at Debre Tabor General Hospital chronic diseases follow-up clinic, Northwest Ethiopia | Excluded | It focusses on KAP rather than barriers and facilitators toward diabetic’s self-care practice |
