# Supplementary material for: Exploring barriers and enablers to diabetes self-care practice in Ethiopia, 2025: A qualitative systematic review
Source: PLoS One. 2026 Apr 10;21(4):e0346867. doi: 10.1371/journal.pone.0346867 (PMC13068285; doi:10.1371/journal.pone.0346867)
Supplement: S2 File — (DOCX) [file pone.0346867.s002.docx]

S2. PRISMA 2020 checklist demonstrating adherence to the Preferred Reporting Items for Systematic Reviews and Meta-Analyses (PRISMA) 2020 guidelines and the PRISMA Extension for Qualitative Evidence Synthesis (PRISMA-QS) on Exploring Barriers and Enablers to Diabetes Self-Care in Ethiopia, 2025**.**

| **Item No.** | **Checklist Item** | **Description** | **Page Number** |
| --- | --- | --- | --- |
| 1 | Title | The title clearly identifies the study as a qualitative systematic review of diabetes self-care practices in Ethiopia. | Page 1 |
| 2 | Abstract | A structured abstract summarizes the background, methods, results, conclusion, and recommendations. | Page 2 |
| 3 | Rationale | The introduction explains the need for a synthesis of qualitative evidence on diabetes self-care due to its growing burden and fragmented understanding in Ethiopia. | Pages 1–2 |
| 4 | Objectives | The objective is to explore and synthesize qualitative evidence on barriers and enablers of diabetes self-care practices in Ethiopia. | Page 3 |
| 5 | Eligibility Criteria | Inclusion and exclusion criteria were defined using the SPIDER framework, focusing on qualitative and mixed-method studies conducted in Ethiopia. | Page 4 |
| 6 | Information Sources | Multiple databases were searched including PubMed, Scopus, CINAHL, Web of Science, and Google Scholar. | Page 4 |
| 7 | Search Strategy | A detailed search strategy using keywords, MeSH terms, and Boolean operators was applied across all databases. | Page 4 |
| 8 | Selection Process | Two reviewers independently screened studies with disagreements resolved through consensus, ensuring methodological rigor. | Page 5 |
| 9 | Data Collection Process | Data extraction was conducted using a structured form capturing study characteristics and key findings. | Page 6 |
| 10 | Data Items | Data items extracted included setting, participants, study design, themes, and quotes relevant to diabetes self-care. | Page 6 |
| 11 | Study Risk of Bias Assessment | The CASP Qualitative Checklist was used to assess study quality, focusing on methodology, ethics, and reflexivity. | Page 6 |
| 12 | Effect Measures | Effect measures are not applicable in qualitative synthesis, which emphasizes thematic interpretation over statistical effect sizes. | Page 8 |
| 13 | Synthesis Methods | Thematic synthesis was applied involving line-by-line coding, development of descriptive themes, and generation of analytical themes. | Pages 6–7 |
| 14 | Reporting Bias Assessment | The review addressed reporting bias by searching across multiple databases and tracking references of included studies. | Page 8 |
| 15 | Certainty Assessment | GRADE-CERQual was used to assess confidence in the findings based on coherence, adequacy, relevance, and methodological limitations. | Page 12 |
| 16 | Study Selection | Study selection is documented using a PRISMA flow diagram detailing the number of records screened and included. | Page 5, Figure 1 |
| 17 | Study Characteristics | Characteristics of the included studies such as study setting, design, and themes are summarized in a table. | Pages 7–8 |
| 18 | Risk of Bias in Studies | Risk of bias was evaluated using CASP with findings showing most studies met quality standards, though some lacked reflexivity. | Page 8 |
| 19 | Results of Individual Studies | Each study’s barriers and enablers are presented with supporting quotes and contextual details. | Pages 9–10 |
| 20 | Results of Syntheses | Synthesized themes of barriers and facilitators were developed based on thematic analysis of all included studies. | Pages 10–13 |
| 21 | Reporting Biases | Some discussion of publication bias is present, although grey literature was not fully explored. | Page 4 |
| 22 | Certainty of Evidence | The certainty of evidence was assessed with GRADE-CERQual; most themes had high or moderate confidence ratings. | Page 12 and Table 5 |
| 23 | Discussion | The discussion places the findings in context with local and international evidence, highlighting key insights. | Pages 14–17 |
| 24 | Limitations of Evidence | The review acknowledges limited representation of rural areas and reporting gaps in ethical procedures. | Page 17 |
| 25 | Limitations of Review Process | Limitations include exclusion of non-English and grey literature, which may affect comprehensiveness. | Page 17 |
| 26 | Implications | Practical recommendations are made for health systems, education, and policy to enhance diabetes self-care. | Pages 17–18 |
| 27 | Registration and Protocol | The protocol was registered in PROSPERO (CRD420251033692), demonstrating commitment to methodological transparency. | Page 3 |
