# Supplementary material for: Exploring barriers and enablers to diabetes self-care practice in Ethiopia, 2025: A qualitative systematic review
Source: PLoS One. 2026 Apr 10;21(4):e0346867. doi: 10.1371/journal.pone.0346867 (PMC13068285; doi:10.1371/journal.pone.0346867)
Supplement: Attachment — Submitted filename: comm.docx [file pone.0346867.s004.docx]

Section-by-Section Comments

1. Study Design

Strong introduction that explicitly states the review type and the reporting standards used.

Consider reducing repetition regarding “transparency, rigor, reproducibility”—one phrase is sufficient.

You may add a brief rationale for choosing a qualitative synthesis (e.g., need for contextual understanding of lived experiences).

2. Aim of the Study

Clear and concise.

Consider adding what the synthesis contributes beyond individual studies (e.g., informing clinical practice, policy, or program development).

3. Review Protocol

Well-written and demonstrates strong methodological accountability.

Consider shortening the paragraph by removing repeated phrases about “methodological integrity.”

The PROSPERO registration number and date are appropriately stated.

4. Search Strategy

Very detailed and technically sound.

Consider reorganizing into:

Databases

Timeframe

Search terms

Supplementary search (citation tracking)

The Boolean terms are appropriate, but ensure that the exact query in Table 1 matches this description.

Phrase “continued to be updated until submission” is good but could be framed more formally, such as “updated prior to manuscript submission.”

5. Inclusion and Exclusion Criteria

Excellent use of the SPIDER framework; clear articulation of each component.

Consider tightening the paragraph by removing overly detailed explanations of each SPIDER element; you may keep the detailed descriptions in Table 1.

The section on grey literature is strong but may benefit from separating the prose into shorter sentences.

Good exclusion criteria; ensure that you explicitly state that mixed-methods studies must have extractable qualitative findings.

6. Search and Screening Process

Clear and consistent with PRISMA guidance.

Good description of dual screening and conflict resolution.

Include whether a pilot screening was performed (optional but strengthens rigor).

The timeline for data extraction and synthesis is helpful but could be moved to a “Review Timeline” subsection or appendix.

7. Data Extraction

Well-structured and consistent with qualitative review standards.

Mention if the extraction form was adapted from JBI or developed by the research team.

Including “illustrative quotations” is appropriate, but ensure the synthesis explicitly uses or references them later.

8. Researcher Reflexivity

Very strong reflexivity section—often missing in qualitative reviews.

Consider briefly summarizing how reflexivity influenced decision-making (e.g., refinement of themes).

You may shorten slightly by removing repeated references to “bias” and “positionality.”

9. Data Synthesis

Correct application of Thomas and Harden’s thematic synthesis method.

Consider specifying the software used for coding, if applicable (e.g., NVivo, ATLAS.ti, manual).

Clarify whether coding was performed independently by two reviewers or jointly from the start.

The collaborative refinement of themes is well described.

10. Quality Appraisal

Good application of CASP and explanation of how results informed interpretation.

The note on not calculating kappa is acceptable, but you may clarify whether calibration exercises were conducted before independent appraisal.

Reporting the distribution of high, moderate, and low-quality studies is excellent and aligns with good practice.

Ensure Table 3 aligns with the narrative.

11. Assessment of Confidence in the Evidence

Strong explanation of CERQual and its components.

You may add a sentence explaining how CERQual findings were integrated into the results section (e.g., “CERQual ratings are presented alongside each synthesized theme”).

Ensure that justifications for each finding are presented in a separate appendix or table.

12. the included eleven studies were with different objective

Summary of Key Strengths

Clear alignment with PRISMA, PRISMA-QS, JBI standards.

Strong methodological transparency and reflexivity.

Comprehensive search strategy and explicit inclusion criteria.

Appropriate use of thematic synthesis and CERQual.

Rigorous dual-review processes.

Priority Areas for Improvement

Reduce minor redundancies to increase conciseness.

Clarify reviewer roles in coding and appraisal (independent vs. collaborative).

Consider reorganizing the Search Strategy and SPIDER criteria for smoother readability.

Move detailed timelines to an appendix to streamline the Methods narrative.
