# Supplementary material for: Exploring barriers and enablers to diabetes self-care practice in Ethiopia, 2025: A qualitative systematic review
Source: PLoS One. 2026 Apr 10;21(4):e0346867. doi: 10.1371/journal.pone.0346867 (PMC13068285; doi:10.1371/journal.pone.0346867)
Supplement: Attachment — Submitted filename: Response to Editor and Reviewer Comments.docx [file pone.0346867.s003.docx]

**Response to Editor and Reviewer Comments**

**Manuscript ID:** PONE-D-25-20746 **Title:** *Exploring Barriers and Enablers to Diabetes Self-Care Practice in Ethiopia, 2025: A Qualitative Systematic Review*

Dear Dr. Aslam and Editorial Team,

We sincerely thank you for reviewing our manuscript and providing detailed, constructive feedback. Your comments were extremely valuable in improving the methodological rigor, clarity, and transparency of our work. We have carefully revised the manuscript in line with each point raised. Below, we provide a detailed, point-by-point response, with reviewer/editor comments reproduced in **bold** and our responses directly underneath.

**Point-by-Point Responses**

**1. The Methods describe a search for “palliative care services” rather than diabetes self-care. It creates confusion to the reader.**

*Response:* We thank the editor for noting this error. This was a leftover phrase from a previous draft. We have corrected all instances where “palliative care” appeared and replaced them with “diabetes self-care.” The revised *Methods → Search Strategy* section now consistently refers to *diabetes self-care practices* as the focus of the review.

**2. The PROSPERO number is mislabeled as a “clinical trial number,” demonstrating confusion about protocol registration versus interventional trials.**

*Response:* We agree with this observation. We have corrected the labeling in the *Declarations* section. The sentence now reads:

“Systematic review protocol registration: PROSPERO CRD420251033692.”

**3. The manuscript does not state whether you limited your search to English-language publications. Excluding non-English studies without justification risks introducing language bias and undermines comprehensiveness.**

*Response:* We appreciate this important point. We clarify in the *Inclusion and Exclusion Criteria* section that **no language restrictions were applied** during the search. We also added a statement on potential language bias in the *Limitations* subsection of the Discussion.

**4. Although you note that grey literature and dissertations were considered, you do not specify which repositories were searched, nor your search terms or screening methods. This lack of detail renders your grey-literature strategy neither transparent nor reproducible.**

*Response:* We have expanded the *Search Strategy* section to provide full details. Grey literature sources now explicitly include **ProQuest Dissertations and Theses, OpenGrey, Ethiopian institutional repositories, and Google Scholar**. We specify that the same search terms were applied, and screening followed the same procedure as for peer-reviewed studies.

**5. No detail is provided on quality appraisal results or how low-quality studies were handled, despite using CASP and GRADE-CERQual.**

*Response:* We have expanded the *Quality Appraisal* subsection to report results. Of the 11 included studies, **7 were rated high quality, 3 moderate, and 1 low** according to CASP criteria. None were excluded on the basis of quality, but lower-quality studies were given proportionally lower confidence ratings in the GRADE-CERQual assessment. A summary of appraisal results has been added in **Table 2 (appendix)**.

**6. Provide, as an appendix, the complete search strings for each platform (including all Boolean operators, field tags, and limits). Specify dates and any filters applied in each database.**

*Response:* We have prepared **Supplementary File 1** containing the complete search strings for PubMed/MEDLINE, Scopus, CINAHL, Web of Science, and Google Scholar, including Boolean operators, field tags, and final search dates (March 15, 2025). Grey literature sources are also detailed.

**7. Re-run the searches and update the PRISMA flow diagram and study count accordingly.**

*Response:* We re-ran the searches with corrected strings and updated the PRISMA flow diagram accordingly. The revised numbers are as follows: Records identified through database searching: 82, Additional records from other sources (grey literature, Google Scholar, etc.): ----, Records after duplicates removed: 28, Full-text articles assessed for eligibility: 11, Full-text articles excluded (with reasons): 0,and Studies included in qualitative synthesis: 11. An updated PRISMA 2020 flow diagram has been included as Figure 1, and a PRISMA exclusion table ( File 2) lists reasons for exclusion at the full-text stage.

We believe that the revisions made in response to the reviewers’ comments have substantially strengthened our manuscript. We have clarified the search strategy, corrected the PROSPERO registration label, reported language considerations, enhanced transparency of our grey literature search, expanded our quality appraisal reporting, provided complete search strings as supplementary material, and updated the PRISMA flow diagram and exclusion table.

We sincerely hope that these changes address all concerns and that the manuscript will now be considered suitable for publication in *PLOS ONE.*

Thank you again for your careful review and constructive feedback.

Sincerely,
**Sadik Abdulwehab** (on behalf of all co-authors)
